# Secondary Oral Vancomycin Prophylaxis and *Clostridioides difficile* Infection in Children and Young Adults With Cancer: A Retrospective Cohort Study

**DOI:** 10.1093/ofid/ofag365

**Published:** 2026-06-20

**Authors:** Isabela DeJohn, Chunyan Liu, Caitlin Brammer, Maryam Mysorewala, Joshua D Courter, Justin Markham, Soumya Jaiswal, Grant Paulsen, Lara Danziger-Isakov, William R Otto

**Affiliations:** University of Cincinnati College of Medicine, Cincinnati, Ohio, USA; Division of Biostatistics and Epidemiology, Cincinnati Children's Hospital Medical Center, Cincinnati, Ohio, USA; Division of Infectious Diseases, Cincinnati Children's Hospital Medical Center, Cincinnati Ohio, USA; Division of Infectious Diseases, Cincinnati Children's Hospital Medical Center, Cincinnati Ohio, USA; Division of Infectious Diseases, Cincinnati Children's Hospital Medical Center, Cincinnati Ohio, USA; Division of Pharmacy, Cincinnati Children's Hospital Medical Center, Cincinnati, Ohio, USA; Division of Infectious Diseases, Cincinnati Children's Hospital Medical Center, Cincinnati Ohio, USA; Division of Pharmacy, Cincinnati Children's Hospital Medical Center, Cincinnati, Ohio, USA; University of Cincinnati College of Medicine, Cincinnati, Ohio, USA; Division of Infectious Diseases, Cincinnati Children's Hospital Medical Center, Cincinnati Ohio, USA; Department of Pediatrics, University of Cincinnati College of Medicine, Cincinnati, Ohio, USA; Division of Infectious Diseases, Cincinnati Children's Hospital Medical Center, Cincinnati Ohio, USA; Department of Pediatrics, University of Cincinnati College of Medicine, Cincinnati, Ohio, USA; Division of Infectious Diseases, Cincinnati Children's Hospital Medical Center, Cincinnati Ohio, USA; Department of Pediatrics, University of Cincinnati College of Medicine, Cincinnati, Ohio, USA

**Keywords:** *Clostridioides difficile* infection, oral vancomycin prophylaxis, pediatric oncology, recurrent *C difficile*

## Abstract

**Background:**

Secondary oral vancomycin prophylaxis has been reported to reduce rates of recurrent *Clostridioides difficile* infection (CDI) in adult patients, but there are limited data in pediatrics. This study sought to assess the impact of secondary oral vancomycin prophylaxis (OVP) during broad-spectrum antibiotic administration in reducing CDI in children and young adults with cancer.

**Methods:**

This was a retrospective study of children and young adults with cancer with primary CDI episode treated from 2017 to 2024. To determine the causal impact of secondary oral vancomycin prophylaxis during antibiotic administration to prevent CDI in the 6 months after the primary episode, propensity score matching and weighted generalized estimating equations were performed.

**Results:**

The initial cohort included 163 patients who experienced primary CDI. After exclusions, 72 patients received 150 courses of broad-spectrum antibiotics within 6 months following their initial infection. Fifteen patients received 27 episodes of OVP. CDIs were not significantly decreased, occurring in 22 of 123 (17.9%) courses in patients who did not receive OVP and 2 of 27 (7.4%) in courses with prophylaxis. Propensity score matching resulted in 49 broad-spectrum antibiotic episodes from 19 patients, forming 11 matched sets. Receipt of OVP was significantly associated with reduced risk of CDI (adjusted odds ratio, 0.074 [95% confidence interval, .01–.54]).

**Conclusions:**

In this single-center retrospective study of children and young adults with cancer with primary CDI, receipt of OVP was significantly associated with reduced risk of CDI recurrence when receiving broad-spectrum antibiotic therapy. Given the small sample size and likely residual confounding, these findings should be considered hypothesis-generating pending confirmation in larger studies.

## Teaser

We assessed the impact of secondary oral vancomycin prophylaxis on subsequent *Clostridioides difficile* infection (CDI) in the 6 months following the index infection in children and young adults with cancer. Receipt of oral vancomycin prophylaxis was significantly associated with reduced risk of subsequent CDI during episodes of broad-spectrum antibiotic therapy.

## Graphical Abstract

**Graphical Abstract:**
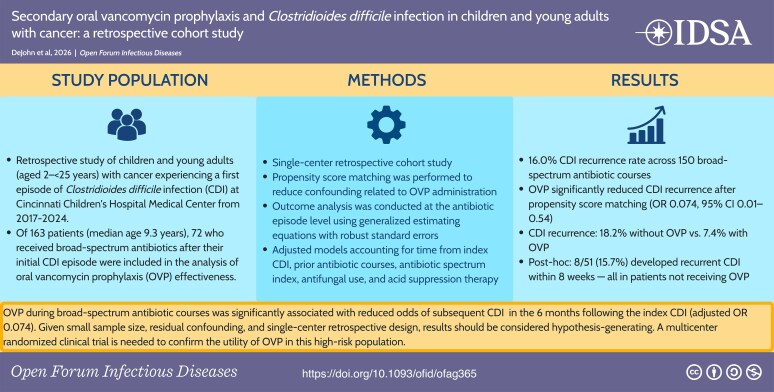
This graphical abstract is also available at Tidbit: https://tidbitapp.io/tidbits/secondary-oral-vancomycin-prophylaxis-and-clostridioides-difficile-infection-in-children-and-young-adults-with-cancer-a-retrospective-cohort-study?utm_campaign=tidbitlinkshare&amp;utm_source=IO

Children and young adults with cancer are at increased risk of *Clostridioides difficile* infection (CDI) and recurrent CDI [1–4]. It has been reported that 5%–11% of hospitalized pediatric cancer patients will suffer from CDI [5, 6], and children and young adults with cancer account for over one-fifth of all pediatric hospitalizations with a CDI diagnosis [7]. Recurrent infection is common in children and young adults with cancer, as 22%–27% of patients are reported to have recurrent CDI within 60 days of their primary infection [8, 9]. In addition to the morbidity and mortality associated with infection, CDI has been linked to delays in chemotherapy, potentially increasing risk of relapse or other adverse cancer outcomes [5, 6, 8]. Given these adverse consequences, great effort has been made to reduce recurrent infection, including use of antibiotic prophylaxis [10]. Secondary oral vancomycin prophylaxis (OVP) has been reported to successfully reduce recurrence rates in adult patients [11–13]. Despite these reports, current clinical practice guidelines discourage use of routine OVP to prevent recurrent CDI due to concerns over microbiome disruption and potential increased risk of recurrent CDI [14, 15]. There are limited data in pediatrics, as only 1 study has examined the use of OVP to prevent recurrent CDI, and there remain key knowledge gaps regarding the optimal patient, dose, and duration of OVP. This study was designed to assess the impact of OVP during broad-spectrum antibiotic administration in reducing CDI recurrence in a contemporary cohort of children and young adults with cancer. We hypothesized that OVP administered during broad-spectrum antibiotic exposure would significantly reduce the risk of subsequent CDI in this high-risk population.

## METHODS

This was a retrospective study of children and young adults with cancer with a first CDI episode treated at Cincinnati Children's Hospital Medical Center (CCHMC) from 1 January 2017 to 31 December 2024. Baseline demographic and clinical data, including age, sex, race/ethnicity, and underlying malignancy, were collected from the electronic medical record. Characteristics of CDI episodes, including severity and treatment, were also collected. Subsequent administration of broad-spectrum antibiotics and oral vancomycin prophylaxis was noted, as well as administration of concomitant medications, including antifungals or acid-suppressing medications. Study data were managed using REDCap [16, 17]. This study was approved by the Institutional Review Board at CCHMC, with a waiver of informed consent (Study ID 2024-0169).

### Patient Cohort

The initial cohort included children and young adults aged 2 years to <25 years with cancer, including hematologic malignancy, solid tumors, or central nervous system tumors. The first episode of CDI had to occur within the study period. Patient medical records and available external documentation were reviewed to identify any prior history of CDI prior to study enrollment. Patients with a confirmed prior episode of CDI were excluded. Patients who had previously undergone hematopoietic cell transplantation were excluded.

For the analysis of the effectiveness of OVP in preventing first recurrence of CDI, patients had to receive broad-spectrum antibiotics in order to be eligible to receive OVP. Resolution of initial infection was documented prior to starting follow-up. Patients who did not receive a course of broad-spectrum antibiotics within 6 months of their initial CDI episode were excluded from the analysis. Antibiotic courses of <48 hours in duration were not included. Patients who were transferred to another institution, were otherwise lost to follow-up, or died within 30 days of initial CDI episode were also excluded.

### Institutional Practices Surrounding CDI

CCHMC utilizes 1-step testing for CDI, using an institutionally developed nucleic acid amplification test designed to detect the gene for *C difficile* toxin. This testing protocol was in place for the entirety of the study period. Institutional protocols recommend testing for *C difficile* if there are at least 3 liquid stools within 24 hours and if there is no alternative explanation for the change in stool consistency (receipt of laxatives in the past 48 hours, change in enteral formula feeds, etc). When testing for *C difficile* infection, it is common practice at our institution to also order multiplex polymerase chain reaction panels for other gastrointestinal pathogens. Stools that are not diarrheal are rejected by the Microbiology Laboratory and testing is not performed. Within the Cancer and Blood Diseases Institute at CCHMC, patients are eligible to receive OVP after suffering an initial episode of CDI. Patients receive OVP dosed at 10 mg/kg (maximum 125 mg per dose) twice daily throughout subsequent courses of systemic broad-spectrum antibiotics. After the index CDI episode, secondary prophylaxis is usually given for future courses of antibiotic therapy.

### Definitions

Subsequent CDI was categorized as a second positive test for *C difficile* toxin within 6 months of the first CDI episode, occurring at least 48 hours after completion of treatment for the initial CDI episode. Definitions of severe and severe complicated CDI are shown in Table 1.

**Table 1.** Definitions of Severe and Severe Complicated *Clostridioides difficile* Infection [18]

Category	Criteria
Severe CDI	Peripheral WBC count >15 000 cells/µL
	Serum creatinine ≥1.5× premorbid level
Severe complicated CDI	Sepsis
	Hypotension requiring vasopressors
	Ileus (radiographic or clinical findings)
	Toxic megacolon
	Intestinal perforation
	Intensive care unit transfer or admission
	Surgery for CDI-related complication
	Death

Abbreviations: CDI, *Clostridioides difficile* infection; WBC, white blood cell.

Antibiotic exposure was quantified using the antibiotic spectrum index (ASI), a validated tool that assigns a numerical score to antibiotics based on their activity against clinically important pathogens [19]. The ASI accounts for the breadth of antimicrobial coverage rather than days of therapy alone, allowing for standardized comparison of antibiotic exposure across a heterogeneous patient population receiving different agents and combinations. The ASI has been reported to be strongly associated with hospital-acquired CDI [20]. For each patient, the total ASI for each day was calculated. For example, a patient receiving cefepime would have an ASI of 6 whereas someone receiving cefepime and metronidazole would have received an ASI of 8. The total daily ASI was then averaged for the duration of the antibiotic course. For this study, broad-spectrum antibiotic exposure was defined as any antibiotic regimen with an average ASI >5, corresponding to the spectrum of ceftriaxone [19]. This threshold was chosen to reflect clinical practice in the oncology setting, where empiric antibiotic regimens narrower in spectrum than ceftriaxone are rarely used.

### Statistical Analysis

Baseline demographic and clinical characteristics for each patient were summarized using descriptive statistics. The clinical characteristics and management of CDI episodes were described. The χ^2^ test or Fisher exact test was used for associations of categorical variables. Wilcoxon rank-sum test was used for differences in continuous variables. The rate of subsequent CDI in the study cohort was calculated at the patient level and at the antibiotic level, where the denominator was the total number of episodes of broad-spectrum antibiotics.

To determine the causal impact of OVP during antibiotic administration to prevent CDI, propensity score matching was performed to reduce confounding related to administration of OVP. A full description of the propensity score–matching process is included in the Supplementary Materials. The final matched data were a combination of both within-cluster and across-cluster matching. Weights were calculated for each matched set where all case weights within a set sum to 1 and all control weights within the same set also sum to 1. Standardized mean differences between cases and controls were calculated to measure the covariate balance (Supplementary Table 1).

After propensity score matching, the outcome analysis was done at the antibiotic episode level using weights to account for variable matching ratios. Robust standard errors were obtained using generalized estimating equations (GEEs) with independence working correlation and clustering by matched set to account for within-set correlation induced by the matching procedure. The unadjusted model only included the treatment in the model. Covariates in the adjusted model included time from index CDI episode, number of prior courses of broad-spectrum antibiotics, the average daily ASI for the broad-spectrum antibiotic course, administration of systemic antifungals (azoles, echinocandins, or amphotericin B), and receipt of proton pump inhibitors or H_2_ blockers. An adjusted model using the whole dataset was performed as a sensitivity analysis.

As a post hoc sensitivity analysis, E-values were calculated for the oral vancomycin prophylaxis exposure to quantify the minimum strength of association that an unmeasured confounder would need to have with both the exposure and outcome to fully explain away the observed association [21].

SAS version 9.4 software (SAS Institute, Cary, North Carolina, USA) was used for descriptive analysis and modeling. R version 4.4.0 software (R Core Team 2024) was used for propensity score analysis. R packages “optmatch” version 0.10.7 were used for propensity score matching [22].

## RESULTS

The initial cohort included 163 children and young adults who experienced a first episode of CDI during the study period (Table 2). The median age was 9.3 years (interquartile range, 4.9–15.7 years). The cohort was majority male and White. The most common underlying cancers were hematologic malignancies (54.0%), followed by solid tumors (36.8%). Approximately one-sixth of patients had relapsed disease at time of the first CDI episode.

**Table 2.** Demographic and Clinical Characteristics of the Cohort

Characteristic	Total^a^ (N = 163)	No Recurrence (n = 113)	Recurrent CDI (n = 44)	*P* Value
Age, y, median (IQR)	9.26 (4.86–15.68)	9.24 (5.07–14.58)	9.4 (3.93–15.18)	.77
Age group				.51
2–5 y	50 (30.7)	32 (28.3)	17 (38.6)	
6–11 y	50 (30.7)	38 (33.6)	10 (22.7)	
12–18 y	50 (30.7)	36 (31.9)	14 (31.8)	
≥19 y	13 (7.98)	7 (6.2)	3 (6.8)	
Sex				.59
Female	69 (42.3)	46 (40.7)	20 (45.5)	
Male	94 (57.7)	67 (59.3)	24 (54.5)	
Race/Ethnicity				.60
Non-Hispanic White	110 (67.5)	71 (62.8)	34 (77.3)	
Non-Hispanic Black	16 (9.8)	13 (11.5)	3 (6.8)	
Middle Eastern	19 (11.7)	16 (14.2)	3 (6.8)	
Hispanic/Latino	4 (2.5)	3 (2.7)	1 (2.3)	
Other or unknown	14 (8.6)	10 (8.8)	3 (6.8)	
Underlying malignancy				.53
Leukemia	66 (40.5)	43 (38.1)	21 (47.7)	
Lymphoma	22 (13.5)	16 (14.2)	5 (11.4)	
Solid tumors	60 (36.8)	42 (37.2)	16 (36.4)	
CNS tumors	15 (9.2)	12 (10.6)	2 (4.5)	
Relapsed cancer at time of CDI episode	28 (17.2)	22 (19.5)	4 (9.1)	.12

Data are presented as No. (%) unless otherwise indicated.

Abbreviations: CDI, *Clostridioides difficile* infection; CNS, central nervous system; IQR, interquartile range.

^a^Includes 6 patients who were lost to follow-up.

Sixteen episodes (9.8%) were classified as severe CDI, most commonly for elevated white blood cell count (13/16). Twenty of 163 (12.3%) episodes were classified as severe complicated CDI. The most common reason for that classification was sepsis (18/20), with 3 patients having hypotension requiring vasopressor support. Ileus (6/20) and intestinal perforation requiring surgical intervention (1/20) were less common. Of the 20 patients that had severe complicated CDI, only 4 patients also were classified as having severe CDI.

The most frequent treatment of primary CDI was oral vancomycin (105/163 [64.4%]), followed by metronidazole (20/163 [12.3%]), with initial metronidazole switched to enteral vancomycin in a few cases (17/163 [10.4%]). Fidaxomicin was rarely used as first-line therapy (3/163 [1.8%]) or following initial treatment with oral vancomycin (6/163 [3.7%]).

**Figure 1.**
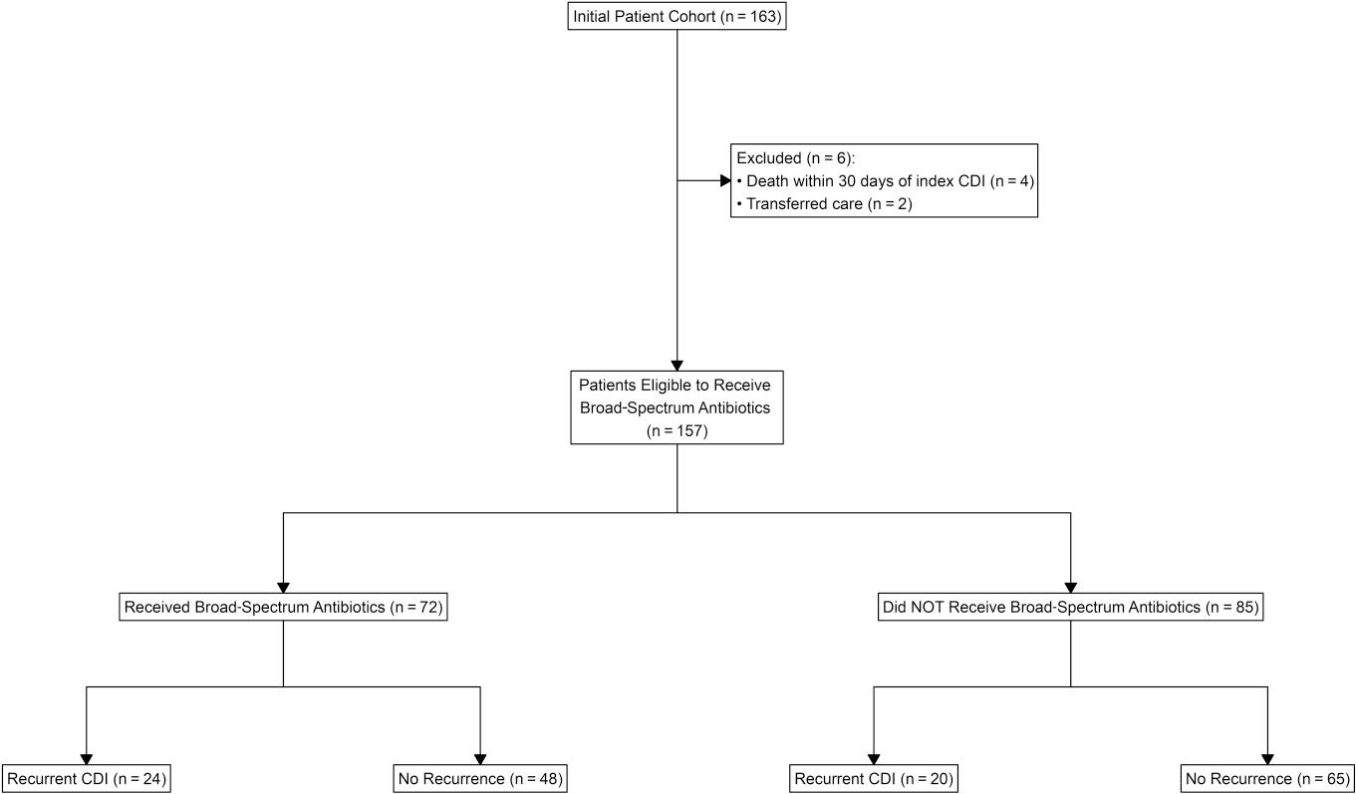
Study cohort. Abbreviation: CDI, *Clostridioides difficile* infection.

Following treatment of the initial CDI episode, 4 patients died within 30 days of completing treatment, while 2 patients transferred to other institutions, leaving 157 children eligible to receive OVP (Figure 1). Eighty-five patients did not receive a course of broad-spectrum antibiotics for >48 hours after their primary CDI episode and were excluded from the analysis of OVP. Notably, 20 of those 85 patients (23.5%) suffered CDI in the 6 months following their initial diagnosis.

A total of 72 patients received 150 courses of broad-spectrum antibiotics within the 6 months following their initial CDI episode, ranging from 1 to 6 episodes. The mean duration of each antibiotic course was 9.1 days (standard deviation [SD], 9.7 days). The average daily ASI was 7.5 (SD, 2.0). Fifteen of 72 patients had a total of 27 episodes of OVP (Table 3). Twenty-four episodes of CDI were observed among the 150 broad-spectrum antibiotic episodes (16.0%; Supplementary Table 2).

**Table 3.** Descriptive of Patient-Level Characteristics by Exposure Groups

Variable	No OVP (n = 57)	Had OVP (n = 15)	Overall (N = 72)	*P* Value^a^
Patient age at first CDI, y, median (IQR)	7.9 (3.7–14.1)	11.2 (6.2–17.0)	8.3 (4.3–14.4)	.18
Sex				.98
Female	23 (40.4)	6 (40.0)	29 (40.3)	
Male	34 (59.6)	9 (60.0)	43 (59.7)	
Patient ethnicity				.11
Hispanic or Latino	1 (1.8)	2 (13.3)	3 (4.2)	
Not Hispanic or Latino	56 (98.2)	13 (86.7)	69 (95.8)	
Patient race				.10
White	35 (62.5)	13 (86.7)	48 (67.6)	
Black	6 (10.7)	…	6 (8.5)	
Asian	1 (1.8)	…	1 (1.4)	
Middle Eastern	11 (19.6)	…	11 (15.5)	
Combined patient race				.12
White	35 (62.5)	13 (86.7)	48 (67.6)	
Non-White	21 (37.5)	2 (13.3)	23 (32.4)	
Patient underlying cancer				.31
Liquid malignancy	26 (45.6)	8 (53.3)	34 (47.2)	
Lymphoma	5 (8.8)	3 (20.0)	8 (11.1)	
Solid tumor	23 (40.4)	3 (20.0)	26 (36.1)	
CNS tumor	3 (5.3)	1 (6.7)	4 (5.6)	
Relapsed cancer	9 (15.8)	2 (13.3)	11 (15.3)	1.00
Severe CDI with the initial episode	6 (10.5)	3 (20.0)	9 (12.5)	.38
Severe complicated CDI with the initial episode	7 (12.3)	2 (13.3)	9 (12.5)	1.00
Patient treatment regimen for first CDI episode				.10
Metronidazole	5 (8.8)	…	5 (6.9)	
Oral vancomycin	36 (63.2)	14 (93.3)	50 (69.4)	
Oral vancomycin taper	1 (1.8)	1 (6.7)	2 (2.8)	
Other	7 (12.3)	…	7 (9.7)	
Metronidazole, then vancomycin	8 (14.0)	…	8 (11.1)	

Data are presented as No. (%) unless otherwise indicated.

Abbreviations: CDI, *Clostridioides difficile* infection; CNS, central nervous system; IQR, interquartile range; OVP, oral vancomycin prophylaxis.

^a^Wilcoxon rank-sum test for continuous variables and χ^2^ or Fisher exact test for categorical variables.

The hybrid propensity score–matching analysis was then enacted. The final matched dataset had 49 broad-spectrum antibiotic episodes from 19 patients, forming 11 matched sets. The number of broad-spectrum antibiotic episode within each matched set ranged from 2 to 10. Fifteen of 19 patients had a total of 27 episodes with exposure to OVP. Six CDI episodes were observed among the 49 broad-spectrum antibiotic episodes (12.2%). Subsequent CDI occurred in 4 of 22 (18.2%) episodes that did not receive prophylaxis compared with 2 of 27 (7.4%) episodes that did receive OVP.

The results of weighted GEEs on this matched data are listed in Table 4. The estimated odds ratio (OR) in the adjusted model was very small (0.074 [95% confidence interval {CI}, .01–.54]). Receipt of oral vancomycin was significantly associated with reduced odds of CDI in the 6 months following the first CDI episode. This effect was not significant in the unadjusted model or the adjusted model using the whole, unmatched dataset. The difficult matching process showed that the cases and controls were not comparable at baseline and justified that propensity score matching was necessary. Therefore, the analysis using the whole data in model 3 is likely biased.

**Table 4.** Results of Weighted Logistic Regression Analysis Using Matched Data

Model Stage	Variable	Odds Ratio (95% CI)	*P* Value
Unadjusted analysis (matched data)	Received oral vancomycin prophylaxis	0.18 (.02–1.69)	.13
Adjusted analysis (matched data)	Received oral vancomycin prophylaxis	0.074 (.01–.54)	.01
	Antibiotic spectrum index	0.55 (.34–.91)	.02
	Number of prior broad-spectrum antibiotic courses	0.36 (.16–.82)	.02
	Received antifungal prophylaxis	6.69 (.53–85.12)	.14
	Received acid suppression	0.51 (.07–3.75)	.51
	Days from index CDI to antibiotic treatment	1.05 (1.01–1.09)	.01
Adjusted analysis (unmatched data)	Received oral vancomycin prophylaxis	0.40 (.08–1.98)	.26
	Antibiotic spectrum index	0.84 (.64–1.10)	.21
	Number of prior broad-spectrum antibiotic courses	1.27 (.77–2.09)	.34
	Received antifungal prophylaxis	1.23 (.47–3.20)	.67
	Received acid suppression	1.34 (.54–3.32)	.53
	Days from index CDI to antibiotic treatment	0.99 (.98–1.01)	.23

Abbreviations: CDI, *Clostridioides difficile* infection; CI, confidence interval.

A post hoc analysis was performed to evaluate the effectiveness of OVP to prevent CDI in the first 8 weeks after the index CDI episode. A total of 51 patients received a course of broad-spectrum antibiotics during that time frame. Six of these 51 patients (11.8%) received OVP; there were no significant differences between groups. Eight of 51 (15.7%) developed recurrent CDI during that time period. All 8 recurrences occurred in patients who did not receive prophylaxis. Due to the small sample size, propensity score matching and outcome analysis was not performed.

## DISCUSSION

We report on a single-center retrospective study of children and young adults with cancer with first-time episodes of CDI and assessed the impact of secondary oral vancomycin prophylaxis during episodes of broad-spectrum antibiotic administration on the development of CDI. Using propensity score matching, receipt of OVP when receiving broad-spectrum antibiotic therapy significantly reduced a second episode of CDI in the 6 months after the index episode.

Our cohort is similar to other published cohorts of children and young adults with cancer with CDI. In this study, severe complicated CDI occurred in 12.3% of patients, while 28.0% suffered CDI within 6 months of the index episode. Willis et al reported on a single-center cohort of 122 children with cancer with CDI [9]. A similar proportion of patients (11.4%) developed complications of CDI treatment. Over one-quarter of patients experienced CDI recurrence within 56 days of the index infection, and nearly half of patients had recurrence of disease within 6 months of the initial CDI episode. Another single-center study of 207 cases of CDI identified severe disease in 20.3% of patients [23]. Rates of recurrence were lower, with recurrence in 15.9% of patients in the first 8 weeks after the index case, and 12.1% with recurrence >8 weeks after the first CDI episode. A larger multicenter study reporting on 627 pediatric cancer and hematopoietic cell transplant patients from hospitals in North America, Europe, and Australia had similar findings [24]. Severe CDI episodes were reported in 70 of 721 cases (9.7%). Recurrent CDI within 40 days occurred in 9.6% of patients, whereas 12.8% of patients had recurrent episodes at any time point.

The association between use of OVP during courses of broad-spectrum antibiotics and reduced risk of CDI is consistent with published literature surrounding use of OVP to prevent recurrent CDI in children, which consists of a single retrospective study of 74 children [25]. In that study, 30 patients received OVP during subsequent courses of broad-spectrum antibiotics [25]. Patients who received OVP had a lower incidence of recurrent CDI (1/30 [3.3%]) than those who did not (11/44 [25.0%]), for a risk difference of −22%. In a multivariable analysis, use of OVP to prevent recurrent CDI was protective (OR, 0.10 [95% CI, .01–.86]).

Notably, recurrence was evaluated within the first 8 weeks after the index episode in the study of Bao et al [25], whereas we evaluated for CDI within the 6 months after the first CDI episode. The longer follow-up window may have allowed for inclusion of episodes that were not true recurrences but rather new infections. Recent data have shown that *C difficile* colonization in children with cancer and hematopoietic cell transplant recipients is a dynamic process and that some patients with history of CDI subsequently have negative testing, possibly indicating that patients have acquisition of new strains [26]. We performed a descriptive analysis of the first 8 weeks after the index CDI, but did not have a large enough sample size to perform a full propensity score–matched analysis to assess the impact of OVP on recurrent CDI. All cases of recurrent CDI occurred in patients who did not receive OVP.

The significant association between OVP and reduced risk of CDI should be viewed cautiously, as residual confounding almost certainly impacts the adjusted model. Both the ASI and the number of prior broad-spectrum antibiotic courses were also significantly associated with a reduction in CDI. However, exposure to any antibiotic has been linked to CDI [27], and antibiotics with broader ASI have also been associated with significantly increased risk of CDI [20]. Similarly, receipt of acid suppression also was associated with reduced risk of CDI in our model despite being associated with increased risk in a large case-control study [28]. We utilized propensity score methods in an attempt to control for confounding related to OVP use, but residual confounding related to different chemotherapeutic treatments, the presence of mucositis, need for abdominal radiation or abdominal surgery, or antibiotic class likely affected this analysis. Similarly, severity classification based on peripheral white blood cell count may be unreliable in children with cancer with chemotherapy-induced neutropenia, potentially resulting in misclassification that could have affected the matching process.

In sensitivity analyses, the E-value for the association between secondary OVP and subsequent CDI was 6.82 for the point estimate and 2.06 for the CI bound, indicating that an unmeasured confounder would need to be associated with both OVP use and subsequent CDI by a risk ratio of at least 2.06 to shift the CI to include the null. Given the small sample size, wide CI, and modest E-value for the confidence bound, residual confounding cannot be excluded, and our findings should be considered hypothesis-generating pending confirmation in larger studies.

Additionally, the data supporting use of OVP to reduce odds of subsequent CDI must be weighed against potential side effects when considering whether to use OVP in clinical practice. Studies in mice models of CDI have shown that mice receiving oral vancomycin are at increased risk of intestinal colonization with pathogens, including vancomycin-resistant enterococci (VRE) and *C difficile* [29, 30]. We were unable to assess for these potential adverse outcomes of OVP, as VRE infections are rare at our institution and VRE screening is not routinely performed in our oncology population. Additionally, microbiome analysis was not routinely performed in this population during the study period, so we were not able to quantify dysbiosis related to use of OVP. These potential adverse outcomes need to be assessed in future trials of OVP.

This study has several limitations. We may have misclassified patients with history of CDI prior to the study period or who developed CDI during the follow-up period. The majority of patients received care only at our institution, and we were able to review outside records for many of the remaining patients, making misclassification less likely. It is possible that patients may have received antibiotics outside of our network or were treated for CDI at other institutions. As our cohort included only children and young adults with cancer, this should be a rare occurrence. Second, our small sample size hampers the analysis and limiting its generalizability. Third, the course-level analysis is subject to potential informative censoring, as follow-up was censored at the start of each subsequent antibiotic course and later courses had less remaining follow-up time within the 6-month observation window, which may have differentially affected observed recurrence rates. Fourth, it is possible that patients with positive *C difficile* testing actually had asymptomatic colonization, with diarrhea due to a different pathogen or noninfectious cause. For this reason, we excluded children <2 years of age. Last, our center uses 1-step molecular testing, so it is possible that patients with positive test results may not have true CDI. This potentially could have biased our results. However, *C difficile* testing is restricted to symptomatic patients, lessening the likelihood of false-positive test results.

In conclusion, administration of oral vancomycin secondary prophylaxis to children and adolescents with cancer during courses of broad-spectrum antibiotic therapy was significantly associated with reduced odds of CDI in the 6 months following a patient's index CDI episode. Given the small sample size in this study and likely residual confounding, it is difficult to make firm conclusions about the generalizability of these findings. A multicenter randomized clinical trial in children is needed to confirm or reject the utility of OVP to prevent CDI recurrence in this high-risk population.

## Supplementary Material

ofag365_Supplementary_Data

## Supplementary Data

Supplementary materials are available at *Open Forum Infectious Diseases* online. Consisting of data provided by the authors to benefit the reader, the posted materials are not copyedited and are the sole responsibility of the authors, so questions or comments should be addressed to the corresponding author.
