# Supplementary material for: Secondary Oral Vancomycin Prophylaxis and Clostridioides difficile Infection in Children and Young Adults With Cancer: A Retrospective Cohort Study
Source: Open Forum Infect Dis. 2026 Jun 20;13(7):ofag365. doi: 10.1093/ofid/ofag365 (PMC13329660; doi:10.1093/ofid/ofag365)
Supplement: ofag365_Supplementary_Data [file ofag365_supplementary_data.docx]

**STROBE Statement — Checklist of Items for Reporting Observational Studies**

Manuscript: Secondary oral vancomycin prophylaxis and Clostridioides difficile infection in children and young adults with cancer: a retrospective cohort study

DeJohn et al.

| **Item** | **No.** | **Recommendation** | **Page/Section Where Reported** |
| --- | --- | --- | --- |
| **Title and abstract** | | | |
| Title and abstract | 1a | Indicate the study's design with a commonly used term in the title or the abstract | Title: "*...a retrospective cohort study*" — design stated explicitly in the title |
|  | 1b | Provide in the abstract an informative and balanced summary of what was done and what was found | Abstract (Background, Methods, Results, Discussion sections) |
| **Introduction** | | | |
| Background/rationale | 2 | Explain the scientific background and rationale for the investigation being reported | Introduction, paragraphs 1–4 (CDI burden in pediatric oncology, OVP adult literature, gap in pediatric data) |
| Objectives | 3 | State specific objectives, including any prespecified hypotheses | Introduction, final paragraph: "We hypothesized that OVP administered during broad-spectrum antibiotic exposure would significantly reduce the risk of subsequent CDI…" |
| **Methods** | | | |
| Study design | 4 | Present key elements of study design early in the paper | Methods, first sentence: "This was a retrospective study of children and young adults with cancer…" |
| Setting | 5 | Describe the setting, locations, and relevant dates, including periods of recruitment, exposure, follow-up, and data collection | Methods: single center (CCHMC), January 1, 2017 – December 31, 2024; 6-month follow-up after index CDI episode |
| Participants | 6a | (Cohort study) Give the eligibility criteria, and the sources and methods of selection of participants. Describe methods of follow-up | Methods – Patient Cohort: age ≥2 to <25 years with cancer, first CDI episode during study period; exclusions described (prior CDI, prior HCT, no broad-spectrum antibiotics, <48 h courses, transfer/loss-to-follow-up, death within 30 days) |
|  | 6b | (Cohort study—matched) Give matching criteria and number of exposed and unexposed | Methods – Statistical Analysis & Supplement: propensity score matching criteria described; 11 matched sets, 27 OVP-exposed vs. 22 unexposed antibiotic episodes |
| Variables | 7 | Clearly define all outcomes, exposures, predictors, potential confounders, and effect modifiers. Give diagnostic criteria, if applicable | Methods – Definitions: subsequent CDI (outcome), OVP (exposure), broad-spectrum antibiotics (ASI >5), severity criteria (Table 1); confounders listed in Statistical Analysis section |
| Data sources/measurement | 8 | For each variable of interest, give sources of data and details of methods of assessment (measurement). Describe comparability of assessment methods if there is more than one group | Methods: electronic medical record (REDCap); one-step institutional NAAT for CDI testing; Antibiotic Spectrum Index described (Institutional Practices and Definitions sections) |
| Bias | 9 | Describe any efforts to address potential sources of bias | Methods – Statistical Analysis: propensity score matching to reduce confounding; Discussion: E-value sensitivity analysis, acknowledgment of residual confounding and potential misclassification bias |
| Study size | 10 | Explain how the study size was arrived at | No formal sample size calculation performed; study is retrospective and includes all eligible patients at a single center over the study period (pragmatic/convenience sample). Noted as a limitation. |
| Quantitative variables | 11 | Explain how quantitative variables were handled in the analyses. If applicable, describe which groupings were chosen and why | Methods – Definitions & Statistical Analysis: ASI averaged over antibiotic course duration; age reported as median (IQR); broad-spectrum threshold defined as ASI >5 (rationale provided) |
| Statistical methods | 12a | Describe all statistical methods, including those used to control for confounding | Methods – Statistical Analysis: descriptive statistics, chi-squared/Fisher's exact, Wilcoxon rank test, propensity score matching (optmatch), weighted GEE with robust SE (independence working correlation, clustering by matched set) |
|  | 12b | Describe any methods used to examine subgroups and interactions | Results: post hoc analysis of first 8 weeks after index CDI episode described; no formal interaction analyses performed |
|  | 12c | Explain how missing data were addressed | Not formally addressed; medical records reviewed for completeness; outside records reviewed for patients receiving partial care externally. |
|  | 12d | (Cohort study) If applicable, explain how loss to follow-up was addressed | Methods – Patient Cohort & Figure 1: patients who died within 30 days (n=4), transferred care (n=2), or did not receive broad-spectrum antibiotics within 6 months (n=85) were excluded; course-level analysis censored at start of subsequent antibiotic course (acknowledged as limitation) |
|  | 12e | Describe any sensitivity analyses | Methods – Statistical Analysis: (1) adjusted model using whole unmatched dataset; (2) E-value calculation for unmeasured confounding; (3) post hoc analysis restricted to first 8 weeks |
| **Results** | | | |
| Participants | 13a | Report numbers of individuals at each stage of study | Results paragraph 1 and Figure 1: 163 initial cohort → 157 eligible → 72 received broad-spectrum antibiotics → 49 in matched dataset |
|  | 13b | Give reasons for non-participation at each stage | Figure 1 and Results: 4 died within 30 days, 2 transferred care, 85 did not receive qualifying broad-spectrum antibiotics |
|  | 13c | Consider use of a flow diagram | Figure 1 (flow diagram provided) |
| Descriptive data | 14a | Give characteristics of study participants and information on exposures and potential confounders | Table 2 (full cohort demographics and CDI characteristics); Table 3 (patient-level characteristics by OVP exposure group); Results paragraphs 1–3 |
|  | 14b | Indicate number of participants with missing data for each variable of interest | Not formally reported. No missing data described; retrospective EMR-based study. |
|  | 14c | (Cohort study) Summarise follow-up time | Methods and Results: follow-up defined as 6 months after index CDI episode; mean antibiotic course duration 9.1 days (SD 9.7); follow-up time not reported separately per patient |
| Outcome data | 15 | (Cohort study) Report numbers of outcome events or summary measures over time | Results: 24/150 (16.0%) broad-spectrum antibiotic episodes resulted in CDI overall; 2/27 (7.4%) with OVP vs. 22/123 (17.9%) without (unmatched); 2/27 (7.4%) vs. 4/22 (18.2%) in matched dataset |
| Main results | 16a | Give unadjusted and confounder-adjusted estimates and their precision (eg, 95% CI). Make clear which confounders were adjusted for and why included | Table 4: unadjusted model, adjusted model (covariates: time from index CDI, number of prior broad-spectrum antibiotic courses, average daily ASI, systemic antifungals, PPI/H2 blockers); adjusted OR 0.074 (95% CI 0.01, 0.54) |
|  | 16b | Report category boundaries when continuous variables were categorized | Methods – Definitions: ASI threshold >5 (corresponding to ceftriaxone spectrum) defined and rationale provided; age groups reported in Table 2 |
|  | 16c | If relevant, consider translating estimates of relative risk into absolute risk for a meaningful time period | Not reported. Absolute risks given descriptively (event rates per antibiotic course over 6-month follow-up window) |
| Other analyses | 17 | Report other analyses done—eg subgroups, interactions, and sensitivity analyses | Results: (1) sensitivity analysis using whole unmatched dataset (Table 4, Model 3); (2) post hoc analysis restricted to first 8 weeks after index CDI; E-value reported in Discussion |
| **Discussion** | | | |
| Key results | 18 | Summarise key results with reference to study objectives | Discussion, paragraph 1: OVP significantly associated with reduced odds of CDI (OR 0.074) during broad-spectrum antibiotic courses, consistent with study hypothesis |
| Limitations | 19 | Discuss limitations of the study, taking into account sources of potential bias or imprecision. Discuss both direction and magnitude of any potential bias | Discussion, Limitations paragraph: (1) potential misclassification of prior CDI history; (2) small sample size; (3) informative censoring at course level; (4) possible asymptomatic colonization; (5) one-step molecular testing without confirmatory toxin assay; (6) residual confounding |
| Interpretation | 20 | Give a cautious overall interpretation of results considering objectives, limitations, multiplicity of analyses, results from similar studies, and other relevant evidence | Discussion and Conclusion: results considered hypothesis-generating; cautious interpretation due to small sample, residual confounding, wide CI; consistent with Bao et al. (2021) |
| Generalisability | 21 | Discuss the generalisability (external validity) of the study results | Discussion and Conclusion: limited generalisability due to single-center design, small sample; multicenter randomised trial recommended |
| **Other information** | | | |
| Funding | 22 | Give the source of funding and the role of the funders for the present study | Funding Statement: University of Cincinnati College of Medicine Medical Student Scholars Program (to ID); John Hauck Foundation (to LDI). No role of funders in study conduct described. |

** Give information separately for exposed and unexposed groups in cohort studies where applicable. | STROBE v4 (combined cohort/case-control/cross-sectional checklist).*

Propensity Score Matching

As exposure to OVP was recorded at the level of each individual course of broad-spectrum antibiotics, but the baseline covariates for the propensity score model were measured at the patient-level, convergence issues arose in the process of building the propensity score model. Therefore, a hybrid M:N matching method was utilized:

1. Within-cluster matching: Case-patients exposed to OVP for a specific course of antibiotics who also had a course of antibiotics where they did not receive OVP served as their own controls for that specific episode.
2. Across-cluster matching: Case-patients who received OVP for all courses of antibiotics were matched to patients who experienced courses of antibiotic therapy when they did not receive OVP. This includes the patients used in the within-cluster matching process, as they all had at least one course of antibiotics without OVP.

A propensity score model was built using logistic regression, including all pre-specified baseline variables that had non-zero variance in both cases and controls. These variables included age, sex, race/ethnicity, underlying malignancy, whether the patient had relapsed disease, and the severity and treatment of the initial CDI episode. Variables with multiple categories were redefined as binary variables to reduce the impact of small categories. The cases were then matched to the controls using the combination of caliper matching on propensity score and exact matching on the variables with zero variance. This M:N matching protocol was performed on the final dataset (72 patients, 150 courses of antibiotics) to maximize the sample size of the matched data.

First, within-cluster matching was done for 9/15 patients who received OVP. The remaining 6 cases were matched using across-cluster matching, where the pool of controls were all the patients who had episodes of broad-spectrum antibiotics without OVP. Patients who received OVP were matched to controls using caliper matching (20% standard deviation on the propensity score) and exact matching on race/ethnicity, presence of severe complicated CDI in the initial episode, and treatment of the first episode due to their invariability in the case group. Four out of the six cases found matched controls. Another round of similar across-cluster matching was done for the remaining 2 cases, with caliper matching on the propensity score and exact matching on just treatment of the prior CDI episode.

| Supplemental Table 1: Covariate balance checking using standardized difference before and after matching | | |
| --- | --- | --- |
| Variable | Standardized difference before matching (N=150) | Standardized difference after matching (N=49) |
| Age | 0.239 | 0.102 |
| Liquid malignancy | 0.169 | -0.258 |
| Lymphoma | 0.163 | 0.088 |
| Solid tumor | -0.345 | 0.204 |
| CNS tumor | -0.019 | 0 |
| Hispanic or Latino | 0.147 | 0.236 |
| Not Hispanic or Latino | -0.147 | -0.236 |
| White | 0.759 | 0 |
| Non-White | -0.707 | 0 |
| Relapse: No | -0.163 | -0.109 |
| Relapse: Yes | 0.163 | 0.109 |
| Severe, complicated CDI: No | -0.017 | 0.164 |
| Severe, complicated CDI: Yes | 0.017 | -0.164 |
| Severe CDI: No | -0.121 | 0.316 |
| Severe CDI: Yes | 0.121 | -0.316 |
| Male | -0.101 | 0.068 |
| Female | 0.101 | -0.068 |
| Metronidazole | NA | NA |
| Vancomycin | 1.784 | 0 |
| Vancomycin taper | 0.110 | 0 |
| Other | NA | NA |
| Metronidazole, then vancomycin | NA | NA |
| Mean absolute Standardized difference | 0.284 | 0.128 |

| Supplemental Table 2: Description of baseline covariate by CDI recurrent groups at patient-level | | | | |
| --- | --- | --- | --- | --- |
| Variable | No Recurrent CDI (N=48) | Recurrent CDI (N=24) | Overall (N=72) | P value* |
| Patient age at first CDI, median (IQR) | 8.2 (5.0, 15.5) | 8.7 (3.8, 14.2) | 8.3 (4.3, 14.4) | 0.80 |
| Sex |  |  |  | 0.50 |
| Male | 30 (62.5%) | 13 (54.2%) | 43 (59.7%) |  |
| Female | 18 (37.5%) | 11 (45.8%) | 29 (40.3%) |  |
| Patient ethnicity |  |  |  | 1.00 |
| Hispanic or Latino | 2 (4.2%) | 1 (4.2%) | 3 (4.2%) |  |
| Not Hispanic or Latino | 46 (95.8%) | 23 (95.8%) | 69 (95.8%) |  |
| Patient race |  |  |  | 0.69 |
| White | 29 (61.7%) | 19 (79.2%) | 48 (67.6%) |  |
| Black | 4 (8.5%) | 2 (8.3%) | 6 (8.5%) |  |
| Asian | 1 (2.1%) |  | 1 (1.4%) |  |
| Middle Eastern | 9 (19.1%) | 2 (8.3%) | 11 (15.5%) |  |
| Combined patient race |  |  |  | 0.14 |
| White | 29 (61.7%) | 19 (79.2%) | 48 (67.6%) |  |
| Non-White | 18 (38.3%) | 5 (20.8%) | 23 (32.4%) |  |
| Patient underlying cancer |  |  |  | 0.66 |
| Liquid malignancy | 22 (45.8%) | 12 (50.0%) | 34 (47.2%) |  |
| Lymphoma | 5 (10.4%) | 3 (12.5%) | 8 (11.1%) |  |
| Solid tumor | 17 (35.4%) | 9 (37.5%) | 26 (36.1%) |  |
| CNS tumor | 4 (8.3%) |  | 4 (5.6%) |  |
| Whether or not patients underlying cancer has relapsed |  |  |  | 0.09 |
| No | 38 (79.2%) | 23 (95.8%) | 61 (84.7%) |  |
| Yes | 10 (20.8%) | 1 (4.2%) | 11 (15.3%) |  |
| Did patient have severe CDI with their initial episode |  |  |  | 0.71 |
| No | 41 (85.4%) | 22 (91.7%) | 63 (87.5%) |  |
| Yes | 7 (14.6%) | 2 (8.3%) | 9 (12.5%) |  |
| Did patient have severe complicated CDI with their initial episode |  |  |  | 1.00 |
| No | 42 (87.5%) | 21 (87.5%) | 63 (87.5%) |  |
| Yes | 6 (12.5%) | 3 (12.5%) | 9 (12.5%) |  |
| Patient treatment regimen for first CDI episode |  |  |  | 0.62 |
| Metronidazole | 4 (8.3%) | 1 (4.2%) | 5 (6.9%) |  |
| Oral vancomycin | 32 (66.7%) | 18 (75.0%) | 50 (69.4%) |  |
| Oral vancomycin taper | 1 (2.1%) | 1 (4.2%) | 2 (2.8%) |  |
| Other | 4 (8.3%) | 3 (12.5%) | 7 (9.7%) |  |
| Metronidazole then vancomycin | 7 (14.6%) | 1 (4.2%) | 8 (11.1%) |  |
| * Wilcoxon rank sum test for continuous variables and Chi-square or Fisher's exact test for categorical variables. | | | | |
